# Relapsed Childhood Acute Lymphoblastic Leukemia: A Single-Institution Experience

**DOI:** 10.7759/cureus.9238

**Published:** 2020-07-17

**Authors:** Pham Nguyen Tuong, Tran Kiem Hao, Nguyen Thi Kim Hoa

**Affiliations:** 1 Oncology, Hue Central Hospital, Hue, VNM; 2 Pediatrics, Hue Central Hospital, Hue, VNM

**Keywords:** relapse, acute lymphoblastic leukemia, treatment

## Abstract

Background

Even though the treatment outcomes of childhood acute lymphoblastic leukemia (ALL) have improved recently, relapse of the disease still remains a challenge in developing countries. This study aims to analyze the incidence of relapse and survival rates in childhood ALL.

Methods

A retrospective study of 156 children with de novo ALL between 2012-2018 was conducted. Data on age, gender, relapse type, and relapse time were analyzed.

Results

A total of 26 (16.7%) patients experienced relapse, with a male-to-female ratio of 2.71:1. The relapse rate in the high-risk group was 1.6 times greater than that in the standard-risk group (61.5% vs. 38.5%). The median time from diagnosis to relapse was 29.3 months (38.5% in the early stage, 26.9% in the intermediate, and 34.6% in the late stage). The most common relapse site was bone marrow (38.5%), followed by the isolated central nervous system (CNS, 23.1%) and CNS plus bone marrow (23.1%); the least common site was testicle with or without bone marrow or CNS (15.2%). The median post-relapse survival time was 7.5 months.

Conclusion

Modification of the protocol to use escalated methotrexate dose and providing new therapies such as stem cell transplantation can improve the overall survival rates in relapsed ALL patients.

## Introduction

Acute lymphoblastic leukemia (ALL) is the most common malignant disease in children. It accounts for one-fourth of all childhood cancers and 72% of all cases of childhood leukemia. Its incidence is estimated to be two to five per 100,000 children. The peak incidence of ALL occurs between the ages of two to five. With the recent advances in chemotherapy, hematopoietic stem cell transplant, and supportive care, the long-term survival in childhood ALL has increased to 85-90%. The most important prognostic factors for determining post-relapse survival rate are relapse site (bone marrow, isolated extramedullary), the timing of relapse (early or late), and the phenotype of the disease [1-3].

Hue Central Hospital, one of the three biggest hospitals with 3,000 beds in Vietnam, plays a vital role in treating childhood ALL in the central region of Vietnam. Since 2008, ALL patients have been treated based on the Children's Cancer Group's (CCG) modified 1882 and 1881 protocols. We carried out this research to analyze the incidence of relapse and survival rate in childhood ALL treated at Hue Central Hospital between January 2012 and April 2018. We believe our findings will contribute to further improving the treatment outcomes in children with ALL.

## Materials and methods

We reviewed the medical records of 156 new patients who were under 16 years of age and diagnosed with ALL. These patients were registered at Hue Pediatric Center, Hue Central Hospital, from January 1, 2012, to April 30, 2018. All patients had received the same treatment as per the modified CCG 1882 and 1881 protocols (Tables 1, 2).

**Table 1:** Treatment regimen for standard-risk ALL (modified CCG-1881) *Patients with central nervous system disease at diagnosis only; **Patients without central nervous system disease at diagnosis will not receive intrathecal therapy on days 14 and 21 ALL: acute lymphoblastic leukemia; CCG: Children's Cancer Group

Drugs	Dose and regimen
1. Induction (1 month)	
Vincristine	1.5 mg/m^2^ (max 2 mg) - days 0, 7, 14, and 21
Dexamethasone	6.0 mg/m^2^/day - days 0-27
L-asparaginase	6,000 IU/m^2^ for 9 doses (3 times weekly) starting on day 2-4
Intrathecal methotrexate	8 mg (age 1 to less than 2 years), 10 mg (age 2 to less than 3 years), 12 mg (older than 3 years) - days 0, 7^*^, 14, and 21^*^
2. Consolidation (1 month)	
Vincristine	1.5 mg/m^2^ (max 2 mg) - days 0, 7, 14, and 21
6-mercaptopurine	75 mg/m^2^/day - days 0-27
Intrathecal Methotrexate	On days 0, 7, 14^**^, and 21^**^
3. Interim maintenance (56 days)	
Vincristine	1.5 mg/m^2^ (max 2 mg) - days 0 and 28
Methotrexate	20 mg/m^2^ - days 7, 14, 21, 28, 35, 42, and 49
6-mercaptopurine	75 mg/m^2^ - days 0-55
Dexamethasone	6 mg/m^2^/day - days 0-4 and 28-32
Intrathecal methotrexate	Once on day 0
4. Delayed intensification (49 days)	
First phase	
Vincristine	1.5 mg/m^2^ (max 2 mg) - days 0, 7, and 14
Dexamethasone	10 mg/m^2^ - days 0-20, then taper over 7 days
L-asparaginase	6,000 U/m2 for 6 doses (3 times weekly) starting on day 2-4
Doxorubicin	25 mg/m^2^ - days 0, 7, and 14
Second phase	
Cyclophosphamide	1,000 mg/m^2^ - day 28
6-mercaptopurine	75 mg/m^2^/day - days 28-41
Cytosine arabinoside	75 mg/m^2^/day - days 29-32 and 36-39
Intrathecal methotrexate	On days 28 and 35
5. Maintenance (84-day cycles; 20 months)	
Vincristine	1.5 mg/m^2^ (max 2 mg) - every 28 days: days 0, 28, and 56
Dexamethasone	6 mg/m^2 ^- days 0-4, 28-32, and 56-60
6-mercaptopurine	75 mg/m^2^/day - days 0-83
Methotrexate	20 mg/m^2^ on days 7, 14, 21, 28, 35, 42, 49, 56, 63, 70, and 77
Intrathecal methotrexate	Once on day 0 of each course

**Table 2:** Treatment regimen for higher-risk ALL (modified CCG-1882) ^¥^Patients with CNS disease at diagnosis only; ^ϕ^­­Patient without CNS disease at diagnosis will not receive intrathecal therapy on days 14 and 21; ^§^Doses escalated for ANC of >2,000 and platelet count of ≥100,000 ALL: acute lymphoblastic leukemia; CCG: Children's Cancer Group; CNS: central nervous system; ANC: absolute neutrophil count

Drugs	Dose and regimen
1. Induction	
Vincristine	1.5 mg/m^2^ (max 2 mg) - days 0, 7, 14, and 21
Prednisolone	60 mg/m^2^/day - days 0-27
L-asparaginase	6,000 IU/m^2^ for 9 doses (3 times weekly) starting on day 2-4
Daunorubicin	25 mg/m^2^/day - days 0, 7, 14, and 21
Intrathecal methotrexate	8 mg (age 1 to less than 2 years), 10 mg (age 2 to less than 3 years), 12 mg (older than 3 years, 12 mg) - days 7^¥^, 14, 21^¥^, and 28
Intrathecal - cytosine arabinoside	30 mg (age 1 to less than 2 years), 50 mg (age 2 to less than 3 years), 70 mg (older than 3 years) – day 0
2. Consolidation (9 weeks)	
Cyclophosphamide	1,000 mg/m^2^ - days 0 and 28
6-mercaptopurine	75 mg/m^2^ - days 0-13 and 28-41
Cytosine arabinoside	75 mg/m^2^/day x 16 doses - days 0-3, 7-10, 28-31, 35-38
Vincristine	1.5 mg/m^2^ (max 2 mg) - days 14, 21, 42, and 49
L-asparaginase	6,000 IU/m^2^ x 12 doses (Monday, Wednesday, Friday) - beginning day 14 (±1 day) and day 42 (±1 day)
Intrathecal methotrexate	On days 0, 7, 14^ϕ^, and 21^ϕ^
3. Interim maintenance (2 months)	
Methotrexate	100 mg/m^2^ - days 0, 10, 20, 30, and 40
Vincristine	1.5 mg/m^2^ (max 2 mg) - days 0, 10, 20, 30, and 40
L-asparaginase	15,000 IU/m^2^ - days 1, 11, 21, 31, and 41
Intrathecal methotrexate	Days 0, 20, and 40
4. Delayed Intensification (2 months)	
Vincristine	1.5 mg/m^2^ (max 2 mg) - days 0, 7, 14, 42, and 49
Dexamethasone	10 mg/m^2^/day - days 0-20
L-asparaginase	6,000 IU/m^2^ x 6 doses - (Monday, Wednesday, Friday) days 3-14, and (Monday, Wednesday, Friday) days 42-53
Doxorubicin	25 mg/m^2^ - days 0, 7, and 14
Cyclophosphamide	1,000 mg/m^2^ - day 28
6-mercaptopurine	75 mg/m^2^/day - days 28-41
Cytosine arabinoside	75 mg/m^2^/day - days 29-32 and 36-39
Intrathecal methotrexate	Days 29 and 36
5. Maintenance [12-week (84-day) cycles]	
Vincristine	1.5 mg/m^2^ (max 2 mg) - days 0, 28, and 56
Prednisolone	40 mg/m^2^/day - days 0-4, 28-32, and 56-60
Methotrexate	20 mg/m^2^/week^§^ - day 7, 14, 21, 28, 35, 42, 49, 56, 63, 70, and 77
6-mercaptopurine	75 mg/m^2^/day^§^ - days 0-83
Intrathecal methotrexate	Day 0 of each cycle

Diagnosis of ALL at presentation was based on the results of bone marrow morphology, where the leukemic blasts were counted for more than 25% in the marrow space. Relapse time was categorized as early, intermediate, and late-stage according to the time from initial diagnosis of <18 months, 18-36 months, and ≥36 months, respectively.

All data were analyzed according to age, gender, relapse timing, relapse period, and relapse site using SPSS Statistics version 20 (IBM, Armonk, NY). Measurement data were described by rate or proportion, and enumeration data were described by means and standard deviations. The Kaplan-Meier method was used for generating survival curves for overall survival. A log-rank test was performed for comparing survival across groups.

## Results

Data relating to 156 new patients with ALL between 2012 to 2018 were collected. Among them, there were 26 relapses, accounting for 16.7% of the total cases. The male-to-female ratio was 2.71:1. The relapse rate in the high-risk group was 1.6 times greater than that in the standard-risk group (61.5% vs. 38.5%); 85.5% of patients achieved remission after the induction phase (Table 3).

**Table 3:** Baseline characteristics of children with relapsed ALL (n=26) ALL: acute lymphoblastic leukemia

Characteristics	N (%)
Gender	
Male	19 (73.1)
Female	7 (26.9)
Risk group classification, n (%)	
Standard	10 (38.5)
High	16 (61.5)
Achieved remission after the induction phase	
Yes	23 (88.5)
No	3 (11.5)

**Figure 1:**
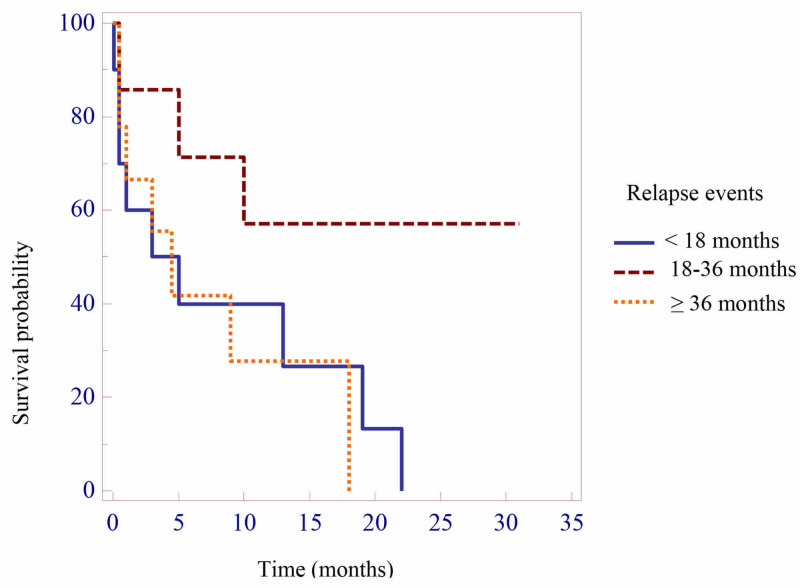
The correlation between relapse events and survival after relapse

The median time from diagnosis to relapse was 29.3 ±18.2 months, of which 38.5% of cases occurred in the early stage, 26.9% in the intermediate, and 34.6% in the late stage. Fourteen (53.8%), six (23.1%), four (15.4%), and two (7.7%) cases relapsed during the maintenance phase, after completing chemotherapy, at delayed intensification phase, and after induction phase (due to treatment abandonment), respectively. The most common relapse site was bone marrow (38.5%), followed by the isolated central nervous system (CNS, 23.1%), and CNS plus bone marrow (23.1%); the least common site was testicle with or without bone marrow or CNS (15.2%). The median post-relapse survival time was 7.5 ±8.3 months, and the three-year post-relapse overall survival rate was 26.9% (Table 4). Patients with intermediate relapse had a better survival rate than those with early relapse and late relapse (Figure 1).

**Table 4:** Characteristics of relapsed patients (n=26)

Characteristics	Value
Relapse timing, n (%)	
Early relapse	10 (38.5)
Intermediate relapse	7 (26.9)
Late relapse	9 (34.6)
Median time to relapse (months)	29.3 ±18.2
Relapse period, n (%)	
Maintenance phase	14 (53.8)
Finished treatment	6 (23.1)
Delayed intensification II	4 (15.4)
Consolidation refused	2 (7.7)
Relapse site, n (%)	
Bone marrow	10 (38.5)
Central nervous system	6 (23.1)
Bone marrow + central nervous system	6 (23.1)
Testicle	2 (7.6)
Testicle + bone marrow	1 (3.8)
Testicle + central nervous system	1 (3.8)
Post-relapse survival	
3-year overall survival rate (%)	26.9
Median survival time (months)	7.5 ±8.3

## Discussion

Our study showed a relapse rate of 16.7%, which is higher compared to findings of studies by Locatelli et al. and Oskarsson et al. [3,4], but lower than in studies by Ali et al. and Nguyen et al., which reported relapse rates of 24.5% and 20.5%, respectively [5,6]. The male-to-female ratio (2.7:1) in this study was similar to some other studies where the relapse rate was higher among males than females; based on these studies, males carry a distinctly poor prognosis factor while females have a better prognosis than males [7,8]. We also noted that the relapse rate in the high-risk group was 1.6 times greater than that in the standard-risk group (61.5% vs. 38.5%). This result was mostly consistent with other studies [6,7].

Among the relapsed cases, three patients (11.5%) did not achieve remission, which was similar to findings by Pizzo and Poplack, which showed that an early response to induction therapy has prognostic value [7]. Regarding the relapse period (53.8%, 23.1%, and 15.4% relapsed during the maintenance phase, after finishing therapy, and delayed intensification II phase, respectively), our results were similar to those of Ali et al., which reported 59.9% of relapsed cases during the maintenance phase [5].

Regarding the relapse site, bone marrow was the leading site and accounted for 38.5%, while the testicle had the lowest rate of relapse (15.3%). Again, these results were similar to the outcomes of Ali et al. and Pizzo and Poplack [7]. The reason for the relatively higher testicle relapse in our study compared to another study could be attributed to our protocol, which was not strong enough to eradicate ALL cells in testicle [9].

At the endpoint (April 2018), 73.1% of relapse patients had passed away while 26.9% of patients were still alive. The median time from relapse to death was 7.5 ±8.3 months, which was shorter compared to other studies. It might be due to the protocol we used. The protocol was not strong enough and was lacking in some tests, such as minimal residual disease to evaluate the response. According to Nguyen et al., overall post-relapse survival rates were higher for patients with isolated CNS relapse (58.7 ±3.2%) than for patients with either isolated (24.1 ±2.1%) or concurrent bone marrow (39.4% ±5.0%) relapses [6].

Regarding the correlation between relapse events and survival after relapse, patients with intermediate relapse had a better survival rate than early relapse (Figure 3). This result appears reasonable. Time to relapse remains the strongest predictor of survival. According to Nguyen et al., estimates of five-years survival rates for isolated marrow relapse in early-, intermediate-, and late-relapsing patients were 11.5 ±1.9, 18.4 ±3.1, and 43.5 ±5.2%, respectively [6]. Van De Berg et al. also showed similar results regarding five-year event-free survival rates for early and late relapses: 12% and 35%, respectively [10].

Recently, several studies have suggested that modification of the protocol to use escalated methotrexate dose and providing new therapies such as stem cell transplantation can improve the overall survival of relapsed patients [11,12].

## Conclusions

In our study, we found that most relapse events in ALL occurred during the maintenance phase and after the completion of chemotherapy. Bone marrow and CNS were the most common relapse sites. According to several recent studies, modification of the protocol to use escalated methotrexate dose and providing new therapies such as stem cell transplantation can improve the overall survival rates in childhood ALL.
